# Knowledge, Attitudes, and Practices Toward Toxoplasmosis Among Veterinary and Human Health Professionals in Central Ethiopia

**DOI:** 10.1155/vmi/4281345

**Published:** 2026-09-02

**Authors:** Derara Bidira, Endrias Zewdu Gebremedhin, Tolesa Negasa, Isayas Asefa Kebede

**Affiliations:** ^1^ School of Veterinary Medicine, Ambo University, P.O. Box 19, Guder, Ethiopia, ambou.edu.et; ^2^ Ethiopian Veterinary Association, P.O. Box 2462, Addis Ababa, Ethiopia; ^3^ College of Veterinary Medicine and Agriculture, Addis Ababa University, P.O. Box 34, Bishoftu, Ethiopia, aau.edu.et

**Keywords:** attitude, Central Ethiopia, health workers, knowledge, practice, toxoplasmosis, veterinary professionals

## Abstract

Toxoplasmosis, caused by the intracellular protozoan *Toxoplasma gondii*, poses significant public health and veterinary challenges worldwide. In Ethiopia, understanding the awareness and behaviors of both animal and human health professionals is vital for the effective prevention and control of diseases. This study assessed the knowledge, attitudes, and practices (KAP) of veterinary and public health professionals regarding the zoonotic importance of toxoplasmosis in Central Ethiopia. A cross‐sectional study was conducted from October 2023 to July 2024 in Holeta and Burayu towns and Addis Ababa city, Central Ethiopia. A total of 411 veterinary and health professionals were selected using a multistage sampling approach and completed a structured questionnaire. Descriptive statistics and ordinal logistic regression analyses were performed using STATA Version 14 to identify factors associated with knowledge, attitudes, and practices scores. The findings showed that 79.3% of respondents had good knowledge, 83% had a positive attitude, and 90% demonstrated good practice. Professionals over 41 years of age were more likely to have a positive attitude compared to those aged 18–30 years (OR = 3.5; 95% CI: 1.1–11.0; *p* = 0.033). Regarding practice, those residing in Addis Ababa had higher odds of good practice than those in Holeta (OR = 2.7; 95% CI: 1.2–6.2; *p* = 0.018), and veterinarians were significantly more likely to exhibit good practice compared to human health professionals (OR = 11.4; 95% CI: 1.5–86.5; *p* = 0.019). However, no factors were significantly associated with knowledge levels. A considerable proportion of veterinary and health professionals in Central Ethiopia demonstrated good knowledge, positive attitudes, and sound practices regarding toxoplasmosis. Strengthening health education and improving healthcare infrastructure are recommended to further enhance awareness and mitigate zoonotic risks.

## 1. Introduction

Livestock plays a central role in the livelihoods of farming communities in Ethiopia, supporting about 60%–70% of the population and contributing significantly to the national economy [1–3]. However, livestock productivity is threatened by numerous diseases, including zoonoses, diseases transmissible from animals to humans that account for over 60% of human infectious diseases globally [4, 5]. Among these, toxoplasmosis, caused by the protozoan parasite *T*. *gondii*, poses significant health risks to both humans and animals [6, 7].

*Toxoplasma gondii* is an obligate intracellular coccidian parasite with a complex life cycle alternating between sexual reproduction in definitive hosts, cats and other felids, and asexual reproduction in intermediate hosts such as humans, livestock, and other warm‐blooded animals [6, 8]. Humans primarily become infected through the ingestion of sporulated oocysts that contaminate soil, food, and water or by consuming undercooked or raw meat containing tissue cysts [9, 10]. Vertical transmission can also occur across the placenta, leading to congenital toxoplasmosis [10–13].

Clinically, most *T. gondii* infections in immunocompetent humans remain asymptomatic; however, approximately 10%–15% of infected individuals may experience symptoms such as lymphadenopathy, mild flu‐like illness, or ocular complications [12, 13]. In immunocompromised persons, including those with AIDS, cancer patients, or transplant recipients, the parasite can cause severe cerebral and systemic disease through the reactivation of latent infections [14, 15]. In pregnant women, primary infection poses serious risks to the fetus, including miscarriage, stillbirth, or congenital abnormalities like hydrocephalus and retinochoroiditis, with severity depending on the gestational stage at infection [12, 16].

In Ethiopia, cultural practices such as the consumption of raw or undercooked meat and the close living proximity between humans and cats increase the risk of *T. gondii* transmission [17, 18]. Cats frequently live outdoors, hunt, and scavenge, behaviors that increase their exposure to the parasite and environmental shedding of infective oocysts [12, 13, 19]. This continuous contamination of the environment sustains the parasite’s life cycle and poses an ongoing risk of infection to humans and livestock [12–14, 16].

Veterinary and human health professionals play a critical role in the detection, prevention, and control of toxoplasmosis and other zoonotic diseases. Their knowledge, attitudes, and practices influence disease surveillance, risk communication, diagnostic decisions, and implementation of preventive measures. However, studies from sub‐Saharan Africa and other regions have reported inadequate awareness and suboptimal preventive practices related to toxoplasmosis among healthcare and veterinary professionals [20–24]. For example, low to moderate knowledge levels and gaps in preventive practices have been documented among healthcare workers and veterinary professionals in countries such as Morocco, Nigeria, and Zimbabwe [4, 7, 19]. These findings highlight the need to strengthen professional capacity for effective toxoplasmosis prevention and control. In Ethiopia, however, evidence regarding toxoplasmosis‐related KAP among veterinary and human health professionals remains limited [17]. Previous studies have primarily focused on specific population groups such as pregnant women [11], community members [24], or individual professional categories [4, 6, 16]. In contrast, the current study assesses both veterinary and human health professionals within the same study setting. Therefore, this study addresses an important evidence gap by providing baseline information on professional capacity for toxoplasmosis prevention and control in Central Ethiopia.

Improving KAP among veterinary and health professionals is essential for reducing the public health burden of toxoplasmosis and minimizing economic losses associated with livestock reproductive problems and reduced productivity. These professionals may also face occupational exposure risks through contact with infected animals, animal tissues, and contaminated materials, particularly veterinarians, veterinary students, and abattoir workers [4, 6, 19]. Strengthening their knowledge and preventive practices can support safer food handling, improved clinical and veterinary management, and coordinated approaches for controlling zoonotic transmission.

Therefore, this study aimed to assess the knowledge, attitudes, and practices of veterinary and health professionals regarding toxoplasmosis in Central Ethiopia. The findings provide evidence to guide targeted professional training, awareness programs, and future zoonotic disease prevention strategies within a One Health framework.

## 2. Methods

### 2.1. Description of the Study Area

**FIGURE 1:**
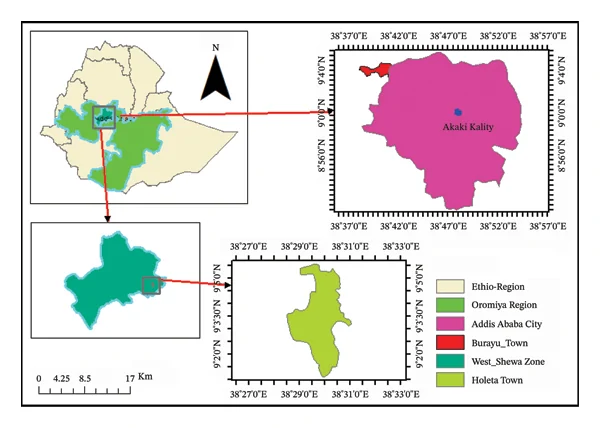
Map of the study area (ArcGIS, 2025).

Holeta town is situated 29 km west of the capital, Addis Ababa, along the main road to Ambo–Nekemte. It has an estimated human population of approximately 42,544 and covers an area of about 61.5 km^2^. Geographically, the town lies at 09°03′00″N latitude and 38°30′00″E longitude, with an elevation of 2391 m above sea level. The annual mean temperature ranges from 11°C to 22°C [25], while the average annual rainfall varies between 900 and 1100 mm. The town is home to an estimated 785 veterinary and health professionals (Figure 1).

Burayu town is located in Sheger City, 15 km west of the capital, Addis Ababa. It has an estimated total population of about 356,416 and covers an area of approximately 66.5 km^2^. Geographically, the town lies at 9°02′30″N latitude and between 38°03′30″E and 38°41′30″E longitude. Positioned in a highland region, Burayu sits at an elevation of 2580 m above sea level. The mean annual temperature is around 14°C, and the average annual rainfall is 1188 mm [26]. The town is home to an estimated 1968 veterinary and health professionals (Figure 1).

Akaki Kality is one of the 11 subcities of Addis Ababa, situated in the southern part of the city, approximately 20 km from the city center (latitude 8.8896°N, longitude 38.7892°E). It serves as the industrial zone of the capital and has an estimated population of over 319,368, covering an area of approximately 61 km^2^, distributed across 13 districts and 91 subdistricts [27]. The subcity is positioned at an elevation of around 2300 m above sea level, with a mean annual temperature of about 15°C and an average annual rainfall of 1200 mm. The subcity is home to an estimated 1750 veterinary and human health professionals (Figure 1). The study population comprised veterinary and human health professionals working in selected subdistricts.

### 2.2. Source and Study Population

The source population for this study comprised all veterinary and human health professionals working in Holeta town, Burayu town, and Akaki Kality subcity of Addis Ababa during the study period.

The study population consisted of veterinary and health professionals who were present in their respective workplaces within these areas at the time of data collection. This group represented a diverse range of age groups, sexes, marital statuses, and educational backgrounds. The target population included veterinary professionals employed in veterinary clinics, pharmacies, and abattoirs, as well as medical professionals working in health posts, hospitals, health centers, and clinics.

### 2.3. Inclusion and Exclusion Criteria

The inclusion criteria for this study were veterinary and health professionals who were willing to participate and provide the required information through the questionnaire, aged 18 or older. Exclusion criteria included respondents who were unwilling to participate, those with hearing impairments preventing them from providing the requested information, and those lacking sufficient time for the questionnaire interview.

### 2.4. Study Design

A cross‐sectional study was conducted from October 2023 to July 2024 to assess the KAP of veterinary and health professionals regarding toxoplasmosis in the Holeta, Burayu, and Akaki Kality subcity of Addis Ababa.

### 2.5. Sample Size Determination and Sampling Techniques

The total number of veterinary and health professionals across the three study areas (Holeta, Burayu, and Akaki Kality subcity of Addis Ababa) was estimated at 17,041. To determine the required sample size for assessing the KAP of these professionals regarding toxoplasmosis, Yamane’s formula [28] was applied:

(1) n=N1+Ne2,

where *n* = required sample size, *N* = population size (17,041), and *e* = margin of error (assumed at 5%).

Using a total population size (N) of 17,041 and a margin of error (e) of 0.05, the calculated sample size was as follows:

*n* = 17,041/[1 + 17,041(0.05^2^)] = 391 participants. To account for a potential 5% nonresponse rate, the sample size was adjusted, resulting in a final sample size of 411 participants.

The three study areas (Holeta town, Burayu town, and Akaki Kality subcity of Addis Ababa) were purposively selected based on the availability of veterinary and human health professionals, healthcare facilities, veterinary services, and the feasibility of data collection. Although purposive selection of study areas may introduce potential selection bias and limit the generalizability of findings beyond the selected settings, this limitation was minimized by applying random sampling procedures at subsequent stages. The required sample size was proportionally allocated across the three study areas: 104 participants from Holeta, 114 from Burayu, and 193 from Akaki Kality subcity. Within the selected administrative units, eligible veterinary and health professionals were selected using simple random sampling from established sampling frames, resulting in the inclusion of 63 veterinary and 348 health professionals.

At the second stage, administrative units were randomly selected: two kebeles from Holeta town and two woredas each from Burayu town and Akaki Kality subcity. These served as the secondary sampling units.

A sampling frame of eligible professionals was generated using official lists obtained from regional and city/town health bureaus, veterinary offices, and professional association registries, ensuring all currently practicing veterinary and human health professionals were included. Professionals who were on leave or retired were excluded.

At the final stage, individual professionals were randomly selected using computer‐generated random numbers, with stratification by profession and study area to ensure proportional representation. Selected professionals were contacted through in‐person visits to their workplaces, and informed consent was obtained before participation. Nonrespondents were replaced by randomly selected alternates from the same professional category and area to maintain proportional representation**.**

This multistage sampling approach combined purposive selection of study areas with random selection procedures at subsequent stages, allowing the inclusion of veterinary and health professionals from diverse public and private institutions. Ethical approval and institutional permissions were obtained from all relevant authorities before data collection.

### 2.6. Data Collection

A structured questionnaire was designed to assess the KAP of veterinary and health professionals regarding toxoplasmosis in the study areas. The tool was developed based on existing literature and pretested on 5% of the sample size (21 professionals) from nearby towns not included in the main study (e.g., Dukem and Sebeta) to ensure clarity, relevance, and comprehension of the items. Only professionals similar in category and demographic characteristics to the study population were included in the pre‐test. Inter‐rater reliability of questionnaire responses was assessed using the Kappa statistic (30), with values above 0.7 indicating substantial agreement, supporting the consistency of the collected data. Content validity was ensured through expert review by epidemiologists, veterinarians, and public health professionals, while construct validity was addressed by structuring questions based on established KAP frameworks and prior literature. Internal consistency of the tool was evaluated using Cronbach’s alpha, with values above 0.7 considered acceptable for each KAP domain.

It was then translated into Afan Oromo and Amharic, the local languages, to ensure clarity and accuracy. Data were collected carefully to obtain all necessary information, and each respondent’s sociodemographic background was documented.

The KAP tool focused on professionals’ knowledge, attitudes, and practices regarding the causes, symptoms, modes of transmission, diagnosis, treatment, and control of toxoplasmosis. It also addressed how people handle and consume animal foods and vegetables, how cat litter is managed, how people interact with the land, the frequency of women’s abortions, and the sources of drinking water.

### 2.7. Data Management and Analysis

All collected data were initially entered into Microsoft Excel 2016 and subsequently imported into STATA Version 14 (StataCorp, College Station, TX, USA) for analysis. Descriptive statistics, including frequencies and percentages, were used to summarize respondent characteristics and KAP scores. Inferential analyses were conducted using univariable and multivariable ordinal logistic regression models.

Because the KAP outcomes were categorized into three naturally ordered levels (knowledge and practice: poor, moderate, and good; attitude: negative, neutral, and positive), ordinal logistic regression (the proportional odds model) was used to account for the ordinal nature of the outcome variables. Ordinal logistic regression was preferred over alternative models, such as multinomial logistic regression, because it incorporates the inherent ordering of the outcome categories and provides a more appropriate framework for analyzing outcomes with meaningful ordinal levels. Variables with a *p* value ≤ 0.25 in the univariable analysis and a variance inflation factor (VIF) < 10 were included in the multivariable models to minimize multicollinearity. The proportional odds assumption for each model was assessed using the Brant test, and no significant violations were detected. Overall model adequacy was evaluated using the omnibus test of model coefficients. Effect estimates were reported as odds ratios (ORs) with corresponding 95% confidence intervals (CIs). All statistical tests were two‐sided, and a significance level of *p* ≤ 0.05 was considered statistically significant.

Knowledge, attitude, and practice (KAP) scores were categorized using established criteria of modified Bloom’s cut points [29]. Knowledge was assessed using 23 questions**,** with each question scored as correct (1) or incorrect (0). The total knowledge score for each participant was calculated by summing all 23 responses, with scores categorized as less than 60% = poor, 60%–80% = moderate, and above 80% = good.

Attitudes were assessed through 11 questions, with each response scored as yes (1) or no/wrong (0). The total attitude score ranged from 0 to 11 and was categorized as < 60% = negative, 60%–80% = neutral, and > 80% = positive toward toxoplasmosis. Practices were assessed through 14 questions using the same scoring approach, and practice scores were categorized using the same thresholds.

Agreement between the categorical KAP variables was assessed using Cohen’s kappa statistic. Kappa coefficients were calculated manually from cross‐tabulated KAP data following standard formulas, and interpretation of agreement levels followed Landis and Koch [30]: *κ* < 0.00 indicates poor agreement; 0.00–0.20, slight; 0.21–0.40, fair; 0.41–0.60, moderate; 0.61–0.80, substantial; and 0.81–1.00, almost perfect agreement. Cohen’s kappa coefficients were reported with corresponding 95% CIs.

## 3. Results

### 3.1. Sociodemographic Characteristics of Study Respondents

A total of 411 veterinary and human health professionals participated in the study. The majority of respondents were human health professionals, and most participants were from Addis Ababa, followed by Burayu. Overall, the study population was predominantly male and consisted mainly of respondents with degree‐level education and moderate work experience. Detailed sociodemographic characteristics of the study participants are presented in Table 1.

**TABLE 1:** Sociodemographic characteristics of study respondents.

Variables	Category	Frequency	Percentage (%)
Service (in years)	Low (1–5)	154	37.5
	Medium (6–15)	218	53.0
	High (above 15)	39	9.5

District	Holeta	104	25.3
	Burayu	114	27.7
	Addis Ababa	193	47.0

Residency	Urban	395	96.1
	Periurban	9	3.0
	Rural	7	1.7

Age (in years)	18–30	143	34.8
	31–40	217	52.8
	Above 41	51	12.4

Sex	Male	264	64.2
	Female	147	35.8

Marital status	Single	86	20.9
	Married	306	74.5
	Widowed	19	4.6

Education level	Diploma	128	31.1
	Degree	239	58.2
	Masters	22	5.4
	Level IV	22	5.4

Religion	Christian	356	86.6
	Muslim	32	7.8
	Wakefata	23	5.6

Occupation	Human health	348	84.7
	Veterinarian	63	15.3

Total		411	100.0

### 3.2. Knowledge, Attitudes, and Practices Regarding Toxoplasmosis

Among the 411 respondents, 96.1% had heard of toxoplasmosis, mainly through education 82.2%, though 77.6% had not received formal training. Over half, 58.4%, correctly identified all at‐risk groups, 89.5% recognized parasites as the cause, 75.7% knew cats are the definitive host, and 86.1% acknowledged high risk in immunocompromised and pregnant individuals (Table 2).

**TABLE 2:** Knowledge of toxoplasmosis among human and veterinary health professionals**.**

Variables	Category	Frequency	Percentage (%)
Have you ever heard of toxoplasmosis?	Yes	395	96.1
	No	16	3.9

If yes, what was your source of information? (Select all that apply)	Education	338	82.2
	Friends	64	15.6
	Training	9	2.2

Have you ever received any formal training on toxoplasmosis?	No	319	77.6
	Yes	92	22.4

Who is affected by toxoplasmosis?	Animal and human	375	91.2
	Only humans	35	8.5
	Do not know	1	0.2

Which groups are at risk of toxoplasmosis? (select all that apply)	All	240	58.4
	Pregnant women	76	18.5
	Immunocompromised individuals	5	1.2
	Cat owners	85	20.7
	Abattoir worker	2	0.5
	Do not know	3	0.7

What is the cause (etiology) of toxoplasmosis? (select all that apply)	Bacteria	40	9.7
	Parasites	368	89.5
	Cold condition	2	0.5
	Hereditary	1	0.2

Is toxoplasmosis present in Ethiopia?	Yes	397	96.6
	No	14	3.6

Which animal is the definitive host for toxoplasmosis?	Dog	2	0.5
	Cattle	9	2.2
	Cat	311	75.7
	Human	51	12.4

What is the risk level of toxoplasmosis to immunocompromised pregnant women?	High	354	86.1
	Low	43	10.5
	Medium	14	3.4

During which trimester of pregnancy is toxoplasmosis most severe?	First	193	47.0
	Second	80	19.5
	Third	138	33.6

What are the clinical signs of toxoplasmosis in the fetus? (select all that apply)	All	204	49.6
	Hydrocephalus	132	32.1
	Retarded brain	43	10.5
	Eye problem	32	7.8

What are the clinical signs of toxoplasmosis in animals? (select all that apply)	Abortion	272	66.2
	In appetence	24	5.8
	Watery diarrhea	37	9.0
	Vomiting	78	19.0

Have you ever encountered a case of toxoplasmosis in a pregnant woman?	Yes	75	18.2
	No	336	81.8

Can drinking raw milk cause toxoplasmosis?	Yes	176	42.8
	No	130	31.6
	I do not know	105	25.5

Can eating raw beef cause toxoplasmosis?	Yes	282	68.6
	No	59	14.4
	I do not know	70	17.0

Can eating raw sheep or goat meat cause toxoplasmosis?	Yes	278	67.6
	No	45	10.9
	I do not know	88	21.4

Can drinking untreated water transmit toxoplasmosis to humans?	Yes	298	72.5
	No	66	16.1
	I do not know	47	11.4

Can contact with cats transmit toxoplasmosis to humans?	Yes	389	94.6
	No	12	2.9
	I do not know	10	2.4

What is used to diagnose toxoplasmosis? (select all that apply)	PCR	68	16.5
	Serology	133	32.4
	Both	194	47.2
	I do not know	16	3.9

Are you familiar with the methods used to diagnose congenital toxoplasmosis before birth?	Yes	255	62.0
	No	42	10.2
	I do not know	114	27.7

Is toxoplasmosis curable after clinical signs appear?	Yes	255	62.0
	No	70	17.0
	I do not know	86	20.9

Can *T. gondii* infection cause vision problems in humans?	Yes	290	70.6
	No	99	24.1
	I do not know	22	5.4

Who is responsible for controlling toxoplasmosis?	Professionals	56	13.6
	Government	17	4.1
	All	338	82.2

Total		411	100.0

In terms of practice, 98.3% respondents supported medical treatment for toxoplasmosis, and 58.2% avoided raw or undercooked meat. Most practiced good hygiene, including handwashing after touching cats (90.0%) and washing vegetables (72.7%), but glove use when handling cat feces was low (22.4%). The majority of respondents (98.8%) reported that local health centers did not diagnose toxoplasmosis, primarily due to a lack of facilities (66.5%) (Table 3).

**TABLE 3:** Human and animal health professionals’ practices for toxoplasmosis prevention and control.

Variables	Category	Frequency	Percentage
What type of treatment is used for human toxoplasmosis? (select all that apply)	Medical treatment	404	98.3
	Traditional treatment	7	1.7

Do you think traditional healers can help in treating toxoplasmosis?	No	215	52.3
	Yes	37	9.0
	I do not know	159	38.7

Do you intend to stop eating raw or undercooked meat because of the risk of *Toxoplasma* infection?	No	172	41.8
	Yes	239	58.2

What type of water do you usually drink at home? (select all that apply)	Bottled	36	8.8
	Spring unboiled	5	1.2
	Spring boiled	11	2.7
	Tap water	359	87.3

Do you wash fruits before eating?	Sometimes	228	55.5
	Always	183	44.5

Do you wash vegetables before eating?	Sometimes	112	27.3
	Always	299	72.7

Do you wash a knife with boiled water after using it to cut raw meat before using it for other foods?	Sometimes	333	81.0
	Always	38	9.2
	Never	40	9.7

Do you allow cats to enter the kitchen?	No	202	49.1
	Yes	209	50.9

Do you use protective gloves when cleaning cat feces?	No	319	77.6
	Yes	92	22.4

Do you cook meat and offal before feeding them to cats?	No	351	85.4
	Yes	60	14.6

Do you wash your hands thoroughly after touching cats and kittens?	No	41	10.0
	Yes	370	90.0

Does your health center diagnose toxoplasmosis?	No	406	98.8
	Yes	5	1.2

If no, reason for no diagnosis	No facility/equipment available	270	66.5
	Lack of attention/priority	136	33.5

What actions do you take to prevent toxoplasmosis?	All (e.g., avoiding raw meat, washing fruits, using gloves when cleaning cat feces, and cooking meat before feeding cats)	410	99.8
	Using cooked vegetables and fruits	1	0.2

Total		411	100.0

Regarding attitudes, 43.3% of respondents considered toxoplasmosis a dangerous disease, and 51.3% disagreed with the misconception that it affects only pregnant women. Most respondents agreed that oral ingestion of contaminated food was the main transmission route and recognized risks associated with raw meat, contaminated milk, and congenital transmission (Table 4).

**TABLE 4:** Health professionals’ attitudes toward toxoplasmosis.

Variables	Category					Total
	Strongly agree	Agree	Neutral	Disagree	Strongly disagree	
Toxoplasmosis is a dangerous disease.	178	191	39	3	0	411
Toxoplasmosis only affects pregnant women.	8	36	118	211	38	
Toxoplasmosis can be transmitted by eating improperly cooked sheep and goat meat.	78	201	105	25	2	
Toxoplasmosis can be transmitted by drinking contaminated raw milk (cow, goat).	121	253	25	12	0	
Toxoplasmosis can be transmitted through blood transfusion.	125	187	38	57	4	
Toxoplasmosis can be transmitted from a pregnant woman to her fetus.	187	181	35	6	2	
Toxoplasmosis can cause miscarriage or stillbirth.	181	190	33	7	0	
Toxoplasmosis can be transmitted through bare‐hand gardening.	25	132	152	93	9	
Women should get tested for toxoplasmosis before pregnancy.	87	222	94	8	0	
The main route of toxoplasmosis transmission is through oral ingestion of contaminated food.	187	144	65	14	1	
Most cases of toxoplasmosis are asymptomatic.	32	230	101	40	8	

### 3.3. Associations Between Knowledge, Attitude, and Practice Scores With Sociodemographic Factors

#### 3.3.1. Knowledge

In univariable analysis, knowledge levels showed no significant differences across service duration, study area, age, sex, marital status, education level, occupation, religion, or residency (Table 5).

**TABLE 5:** Univariable analysis of sociodemographic factors and knowledge levels.

Variables	Category	Knowledge level			Univariable output	
		Poor	Medium	Good	OR (95% CI)	*p* value
Service (in years)	Low (1–5 years)	14	19	121	Ref.	—
	Medium (6–15 years)	20	23	175	1.1 (0.6, 1.8)	0.69
	High (> 15 years)	4	5	30	1.0 (0.4, 2.5)	0.94

Study area	Holeta	18	19	67	Ref.	—
	Burayu	12	11	91	1.1 (0.6, 2.2)	0.76
	Addis Ababa	8	17	168	0.8 (0.5, 1.5)	0.55

Age (in years)	18–30	13	17	113	Ref.	—
	31–40	19	23	175	1.0 (0.6, 1.7)	0.84
	> 41	6	7	38	1.0 (0.5, 2.3)	0.92

Sex	Female	8	15	124	Ref.	—
	Male	30	32	202	1.2 (0.7, 2.0)	0.41

Marital status	Single	12	11	63	Ref.	—
	Married	23	35	248	0.8 (0.4, 1.4)	0.36
	Widowed	3	1	15	1.6 (0.5, 5.3)	0.42

Education level	Diploma	11	16	101	Ref.	—
	Degree	22	27	190	1.1 (0.6, 1.7)	0.80
	Masters	4	3	15	1.6 (0.5, 4.8)	0.44

Occupation	Human health	32	41	275	Ref.	—
	Veterinarian	6	6	51	1.2 (0.5, 2.2)	0.68

Religion	Wakefata	3	2	27	Ref.	—
	Christian	33	43	280	1.2 (0.4, 3.3)	0.77
	Muslim	2	2	19	1.4 (0.5, 3.3)	0.48

Residency	Periurban	2	3	4	Ref.	—
	Urban	35	42	317	1.8 (0.3, 10.0)	0.48

Abbreviations: %, percentage; CI, confidence interval; OR, odds ratio; Ref., reference category..

#### 3.3.2. Attitude

In univariable analysis, respondents over 41 years old were more likely to have positive attitudes (OR = 3.3; 95% CI: 1.1–9.8; *p* = 0.03). However, no significant differences in attitude scores were observed by district, sex, education level, or occupation (Table 6).

**TABLE 6:** Univariable analysis of sociodemographic factors and attitude/practice levels.

Variables	Category	Attitude level			Univariable output		Practice level			Univariable output	
		Negative	Medium	Positive	OR (95% CI)	*p* value	Poor	Medium	Good	OR (95% CI)	*p* value
Service (in years)	Low (1–5 years)	10	16	128	**Ref.**	—	5	8	141	Ref.	—
	Medium (6–15 years)	16	20	182	1.0 (0.6, 1.7)	0.96	13	9	196	0.8 (0.4, 1.6)	0.55
	High (> 15 years)	2	6	31	0.8 (0.3, 1.9)	0.65	1	5	33	0.5 (0.2, 1.4)	0.23

District	Holeta	10	13	81	Ref.	—	10	3	91	Ref.	—
	Burayu	3	12	99	1.9 (0.9, 3.9)	0.07^a^	8	6	100	1.0 (0.4, 2.3)	0.90
	Addis Ababa	15	17	161	1.4 (0.8, 2.6)	0.25	1	13	179	1.9 (0.9, 4.4)	0.10

Age (in years)	18–30	13	18	112	Ref.	—	5	7	131	Ref.	—
	31–40	14	21	182	1.4 (0.8, 2.5)	0.18	12	10	195	0.8 (0.4, 1.6)	0.56
	> 41	1	3	47	3.3 (1.1, 9.8)	0.03^a^	2	5	44	0.6 (0.2, 1.6)	0.30

Sex	Female	15	11	121	Ref.	—	9	6	132	Ref.	—
	Male	13	31	220	1.1 (0.7, 1.9)	0.63	10	16	238	1.1 (0.5, 2.1)	0.85

Marital status	Single	7	10	69	Ref.	—	1	4	81	Ref.	—
	Married	20	31	255	1.2 (0.7, 2.3)	0.50	17	17	272	0.4 (0.2, 1.2)	0.14
	Widowed	1	1	17	2.1 (0.4, 9.8)	0.36	1	1	17	0.5 (0.9, 2.9)	0.45

Education level	Diploma	15	6	130	Ref.	—	6	5	139	Ref.	—
	Degree	15	34	190	0.6 (0.6, 1.1)	0.11	11	15	213	0.7 (0.3, 1.4)	0.26
	Masters	1	2	21	3.2 (0.4, 25.4)	0.26	2	2	18	0.3 (0.1, 1.2)	0.10

Occupation	Human health	24	39	285	Ref.	—	18	21	308	Ref.	—
	Veterinarian	4	3	56	1.7 (0.7, 3.9)	0.20	1	1	61	8.1 (1.1, 60.2)	0.04

Religion	Wakefata	2	5	32	Ref.	—					
	Christian	23	35	298	1.5 (0.5, 4.2)	0.44					
	Muslim	3	2	23	1.0 (0.3, 3.9)	0.90					

Abbreviations: %, percentage; CI, confidence interval; OR, odds ratio; Ref., reference category.

#### 3.3.3. Practice

In univariable analysis, veterinarians were significantly more likely to have good practices than human health professionals (OR = 8.1; 95% CI: 1.1–60.2; *p* = 0.04). No clear associations were observed for district, age, sex, education, or marital status (Table 6).

Before multivariable analysis, all variables from the univariable analysis were checked for collinearity, and no significant issues were detected (all correlation values < 0.7; VIF < 10). Variables with *p* ≤ 0.25 in the univariable analysis were included in the multivariable ordinal logistic regression model.

In the multivariable analysis, study area and occupation were significantly associated with practice, while age and education level were significantly associated with attitude. Respondents over 41 years were more likely to have a positive attitude compared to those aged 18–30 (OR = 3.5; 95% CI = 1.1–11.0; *p* = 0.03). Similarly, respondents from Addis Ababa were more likely to report good practices than those from Holeta (OR = 2.7; 95% CI = 1.2–6.2; *p* = 0.02), and veterinarians were more likely to demonstrate correct practices than human health professionals (OR = 11.4; 95% CI = 1.5–86.5; *p* = 0.02) (Table 7).

**TABLE 7:** Multivariable analysis of sociodemographic factors and attitude/practice scores.

Variables	Category	Attitude level			Multivariable		Variables	Category	Practice level			Multivariable	
		Negative	Medium	Good	OR (95% CI)	*p* value			Poor	Moderate	Good	OR (95% CI)	*p* value
Age	18–30	13	18	112	Ref.	—	Study area	Holeta	10	3	91	Ref.	—
	31–40	14	21	182	1.6 (0.9, 2.8)	0.11		Burayu	8	6	100	1.3 (0.6, 3.0)	0.54
	Above 41	1	3	47	3.5 (1.1, 11.0)	0.03		Addis Ababa	1	13	179	2.7 (1.2, 6.2)	0.02

Education level	Diploma	15	6	130	Ref.	—	Occupation	Human health	18	21	308	Ref.	—
	Degree	15	34	190	0.5 (0.3, 0.9)	0.05		Veterinarian	1	1	61	11.4 (1.5, 86.5)	0.02
	Masters	1	2	21	1.8 (0.2, 14.9)	0.60							

*Note:* Variables with *p* ≤ 0.25 in univariable analysis were included in the multivariable model.

Abbreviations: CI, confidence interval; OR, odds ratio; Ref., reference category.

**FIGURE 2:**
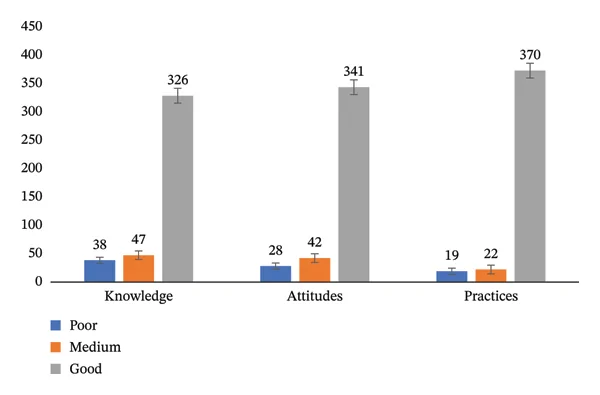
Distribution of knowledge, attitude, and practice levels regarding toxoplasmosis among veterinary and human health professionals (*n* = 411).

Most respondents showed good knowledge (79.3%), positive attitudes (83.0%), and good practices (90.0%), combining both human and animal health professionals (Figure 2).

Cohen’s kappa analysis was conducted to evaluate agreement between categorical KAP variables (Table 8). Knowledge and attitude showed substantial agreement (*κ* = 0.790; 95% CI: 0.716–0.864; *p* < 0.001), while knowledge and practice (*κ* = 0.612; 95% CI: 0.543–0.680; *p* < 0.001) and attitude and practice (*κ* = 0.711; 95% CI: 0.640–0.783; *p* < 0.001) demonstrated substantial agreement. These findings indicate that theoretical knowledge and attitudes are closely aligned, whereas the translation into actual practice is lower, likely reflecting structural constraints, resource limitations, or other practical barriers that hinder consistent implementation of preventive measures.

**TABLE 8:** Cohen’s kappa coefficients for KAP data of 411 respondents.

Comparison	Kappa (*κ*)	95% CI	Std. Err	Z	*p* value	Interpretation
Knowledge vs attitude	0.790	0.716–0.864	0.038	20.89	*p* < 0.001	Almost perfect
Knowledge vs practice	0.612	0.543–0.680	0.035	17.42	*p* < 0.001	Substantial
Attitude vs practice	0.711	0.640–0.783	0.037	19.39	*p* < 0.001	Substantial

## 4. Discussion

### 4.1. Knowledge and Awareness Gaps

This study evaluated the KAP of veterinary and human health professionals regarding the zoonotic significance of toxoplasmosis in Central Ethiopia, focusing on the awareness of clinical signs, transmission pathways, treatment, and prevention strategies. The reliability of the questionnaire, confirmed by substantial inter‐rater agreement (kappa > 0.7), supports confidence in the findings. Understanding toxoplasmosis transmission and clinical features is crucial for effective public health interventions, particularly in resource‐limited settings [17]. The KAP framework suggests that improved knowledge can positively influence attitudes and practices, enhancing disease prevention [20, 31], while public education remains a central tool in zoonotic disease control [17, 32, 33]. The results indicate that most government veterinary and human health professionals possess good knowledge, especially regarding transmission, prevention, and treatment, providing a foundation for future training and policy development.

Despite generally good knowledge, only 22.4% had prior training, and understanding of key aspects, causative agent, high‐risk groups, clinical signs, transmission, preventive measures, cross‐contamination, and vulnerable pregnancy periods remained limited. Preventive practices, including avoiding raw meat and safely handling cats, were inconsistently applied. Attitudes toward toxoplasmosis were generally less favorable, and structural limitations, such as scarce diagnostic facilities, hinder effective disease control [17, 19, 32, 33], suggesting that knowledge alone may not translate into optimal practice.

Most participants (89.5%) correctly identified *T*. *gondii* as the causative agent, and 75.7% recognized cats as the definitive host, showing higher awareness than studies from Yemen, Iran, and Saudi Arabia [33–35]. This difference may reflect variations in professional training, emphasis on zoonotic education, exposure to public health initiatives, regional health priorities, and study methods, highlighting the need for context‐specific, ongoing education programs to sustain and improve professional knowledge.

Regarding transmission routes, 72.5% of professionals recognized untreated water as a potential source, higher than reports by Alrashada et al. [36]. Similar waterborne risks have been reported from Mexico [37]. Such differences likely reflect variations in water sources, treatment practices, public health education, and study populations, highlighting the need for professionals to incorporate waterborne transmission awareness in community guidance.

Knowledge gaps were also evident concerning immunocompromised patients. Few respondents recognized toxoplasmosis as an opportunistic infection, despite its potential to cause severe complications, including fatal encephalitis in HIV patients [37–39]. This indicates the need for targeted training to improve professionals’ understanding of risks in vulnerable populations and enhance preventive and clinical practices.

Significant misunderstandings regarding clinical signs, transmission, and treatment were noted: 19% incorrectly identified vomiting as an animal symptom, 68.6% believed raw beef was a primary source of infection despite sheep, goats, and pigs being the main reservoirs, only a minority knew the disease was treatable after symptom onset, and 24.1% did not recognize ocular complications in humans. These findings emphasize the importance of targeted, on‐the‐job training to strengthen disease recognition, management, and public health outcomes.

In the univariate analysis (Table 5), none of the sociodemographic factors were significantly associated with knowledge. This likely reflects the relatively homogeneous knowledge levels among participants and the small sample sizes in certain subgroups, which may have limited the statistical power to detect significant associations.

### 4.2. Attitudinal Barriers and Sociodemographic Predictors

In this study, 86.6%, 83%, and 90% of animal and human health professionals demonstrated adequate KAP regarding toxoplasmosis, respectively. While overall knowledge was higher than reported in Saudi Arabia [11, 36], Iran [35], and other parts of the world [40], significant gaps remained. Notably, only 33.6% of respondents correctly identified the critical period during pregnancy when maternal infection poses the greatest risk to the fetus. Although transmission rates peak in the third trimester, the most severe fetal outcomes occur during the first trimester due to the thicker placental barrier early in gestation, which gradually thins [37, 41]. These gaps suggest that professional training, access to current information, regional initiatives, and differences in study design or respondent characteristics influence knowledge, emphasizing the need for tailored, ongoing education for professionals.

Multivariable ordinal logistic regression revealed that age and education significantly influenced attitudes toward toxoplasmosis. Older professionals were more likely to hold favorable attitudes, with those above 41 years showing higher odds compared to the 18–30‐year age group (OR = 3.5; 98% CI: 1.1–11.0; *p* = 0.03). This suggests that cumulative professional experience, repeated exposure to zoonotic cases, and ongoing engagement with public health practices strengthen risk perception and preventive behavior.

Education level was also associated with attitude. Respondents with a degree were less likely to hold negative or neutral attitudes than diploma holders (OR = 0.5; 98% CI: 0.3–0.9; *p* = 0.05), indicating that formal undergraduate training reinforces understanding and a preventive mindset. The absence of a significant effect among master’s degree holders (OR = 1.8; 98% CI: 0.2–14.9; *p* = 0.60) likely reflects the small sample size rather than a lack of influence. Collectively, these results highlight that both age and education shape professionals’ perceptions, suggesting that targeted awareness programs for younger or less formally educated staff could strengthen preventive attitudes and practices.

The lack of significant associations between other sociodemographic variables and attitudes implies that general awareness of toxoplasmosis is relatively uniform across different groups, possibly due to standardized training curricula or widespread exposure to public health messaging.

### 4.3. Practice Deficiencies and System‐Level Constraints

Among the 63 animal health workers surveyed, nearly all demonstrated knowledge of the zoonotic nature of *Toxoplasma* infection, including its transmission, treatment, control, and prevention. Despite this awareness, none had diagnosed the disease in animals, reflecting the difficulty of clinical diagnosis and the lack of laboratory facilities in veterinary clinics and regional laboratories. Furthermore, these professionals had never participated in community education regarding the zoonotic risks of toxoplasmosis, the safe handling and consumption of animal products, or the role of raw meat and cat feces in transmitting the disease to pregnant women. This gap highlights insufficient collaboration between the veterinary and medical sectors in promoting community awareness. Strengthening intersectoral collaboration, improving laboratory infrastructure, and providing targeted training for animal health workers on zoonotic diseases are critical steps to enhance early detection and prevention efforts.

Among the 348 human health professionals studied, 86.6% were knowledgeable about zoonotic *Toxoplasma* infection.This is a relatively high level, but itremains suboptimal given its public health significance, particularly for pregnant women and immunocompromised individuals. Most knowledgeable respondents were aware of transmission routes, treatment, and preventive measures; however, nearly all had never diagnosed toxoplasmosis in humans or animals. Reported barriers included limited diagnostic facilities (66.5%) and insufficient attention to the disease (33.5%). These findings indicate that improving laboratory capacity, offering targeted professional training, and integrating toxoplasmosis into routine diagnostic protocols are essential to strengthen disease detection and management.

The study identified persistent risky behaviors despite awareness of *T*. *gondii* risks: 41.8% consumed raw or undercooked meat, 55.5% washed fruits only occasionally, and 81.0% rarely used boiled water to clean knives after cutting raw meat. These practices increase the potential for foodborne transmission and cross‐contamination, highlighting a gap between knowledge and behavior.

The observed discrepancy between relatively high knowledge levels and suboptimal preventive practices suggests that knowledge alone may not be sufficient to achieve sustained behavioral change. Preventive practices are influenced by multiple factors, including availability of resources, access to diagnostic and training facilities, workplace conditions, cultural practices, and individual risk perception. For example, although many professionals recognized the zoonotic importance of toxoplasmosis, behaviors such as consumption of raw or undercooked meat and inadequate handling of cat feces remained common, indicating that established cultural habits and practical constraints may limit the translation of knowledge into action. Therefore, interventions should extend beyond information provision and incorporate practical training, improved workplace support, and context‐specific behavior change strategies to promote effective toxoplasmosis prevention.

Hygienic practices related to cats, the definitive hosts of *T. gondii*, were suboptimal: 77.6% did not use gloves when cleaning cat feces, 85.4% fed cats uncooked meat or offal, and 98.8% reported that their health centers do not diagnose toxoplasmosis. These findings highlight the need for comprehensive training, public health education, and strengthened diagnostic capacity.

Multivariable analysis indicated that both district of residence and occupation were significantly associated with preventive practices. Professionals residing in Addis Ababa were more likely to demonstrate good practices compared to those in Holeta (OR = 2.7; 95% CI: 1.2–6.2; *p* = 0.02), suggesting that urban settings with better infrastructure, access to information, and institutional support facilitate implementation of preventive measures.

The analysis indicated that occupation was a key factor associated with good practice. Veterinarians were substantially more likely to implement appropriate preventive measures than human health professionals (OR = 11.4; 95% CI: 1.5–86.5; *p* = 0.02), likely due to more frequent animal contact and greater familiarity with zoonotic disease prevention protocols. The wide CI reflects limited precision due to the small number of veterinarians, so the result should be interpreted cautiously. However, it underscores the importance of targeted health education, interprofessional collaboration, and practical training for human health professionals, particularly in semiurban areas, to bridge gaps and promote consistent preventive practices. Variables such as age and education did not significantly influence practice, suggesting that structural or systemic factors may play a larger role than individual demographics in shaping preventive behaviors.

This study explored how sociodemographic factors influence the knowledge, attitude, and practice (KAP) of veterinary and health professionals regarding toxoplasmosis. Knowledge levels were generally high and showed no significant variation across demographics, indicating broad awareness. However, differences appeared in attitudes and practices: older professionals and those with higher education, particularly degree holders, tended to have more positive attitudes, likely reflecting both experience and formal training. In terms of preventive behaviors, professionals based in Akaki Kality subcity and those in the veterinary field performed better, likely due to better access to resources and training. These findings highlight the need for targeted interventions aimed at younger, less‐educated professionals and those in periurban areas to strengthen disease prevention efforts.

Cohen’s kappa analysis revealed near‐perfect alignment between knowledge and attitude, indicating that understanding of toxoplasmosis largely informs beliefs and perceptions. In contrast, lower agreement between knowledge and attitude and actual practice suggests that having the right information does not always translate into consistent preventive actions. Practical and structural barriers, such as limited diagnostic facilities, insufficient resources, and high workloads, likely contribute to this gap, restricting the application of knowledge in daily practice.

The results carry significant implications within the One Health framework [31], which emphasizes the interconnectedness of human, animal, and environmental health. Insufficient KAP among veterinary and human health professionals can increase the risk of zoonotic transmission and undermine livestock disease control programs [19–22]. Gaps in awareness and preventive behavior not only threaten human health but also reduce livestock productivity due to reproductive losses, highlighting both economic and public health consequences [7, 31]. Strengthening KAP enhances safe animal handling, minimizes occupational exposure, and improves surveillance and response measures, reinforcing integrated One Health strategies [4]. Well‐informed professionals can thus play a key role in reducing disease transmission, improving food safety, and mitigating health and economic burdens, underscoring the importance of coordinated interventions in Ethiopia.

### 4.4. Limitations of the Study

This study has several limitations that should be considered when interpreting the findings. Its cross‐sectional design precluded causal inferences between sociodemographic variables and KAP outcomes. Reliance on self‐reported responses may have introduced social desirability or recall bias, particularly for behaviors such as hand hygiene, potentially affecting the accuracy of reported knowledge and practices. The relatively small number of veterinary professionals compared to human health professionals, along with limited representation of Muslim and Wakefata respondents, may have introduced bias and confounding, so interpretations based on occupation or religion should be made cautiously.

The study was conducted in selected districts of Central Ethiopia, Holeta, Burayu, and Akaki Kality (Addis Ababa), chosen for access to hospitals, health centers, and veterinary professionals. While this facilitated recruitment, it may have introduced selection bias and limits the generalizability of findings to other towns, particularly rural and semiurban areas. Laboratory‐confirmed exposure to *T*. *gondii* was not assessed, which could have strengthened interpretations of reported practices and associated risks. Moreover, as the study population comprised health professionals, who likely have higher KAP levels than the general community, the results reflect professional rather than community‐level knowledge and practices.

The multistage sampling procedure may have introduced a design effect, potentially increasing variability compared to simple random sampling. Although the sample size was adjusted for nonresponse, the effect of clustering was not formally accounted for, so the findings should be interpreted cautiously regarding generalizability beyond the study population.

Finally, this study did not assess the interconnections between human, animal, and environmental health components within a One Health framework. Future studies incorporating One Health approaches are recommended to provide a more comprehensive understanding of *T*. *gondii* transmission dynamics and to inform integrated intervention strategies.

Despite these limitations, the study provides valuable insights as the first integrated assessment of KAP regarding toxoplasmosis among veterinary and human health professionals in Central Ethiopia. Inclusion of respondents from both urban and periurban settings allowed meaningful comparisons and highlighted specific practice gaps. The use of multivariable ordinal logistic regression strengthened analytical rigor by controlling for potential confounders, while high response rates and a validated, pretested questionnaire support the reliability and applicability of the findings for public health strategies and professional training programs.

## 5. Conclusion

This study demonstrated that veterinary and human health professionals had generally good knowledge of toxoplasmosis, particularly regarding its transmission, treatment, prevention, and control. However, limited experience in diagnosing toxoplasmosis in humans and significant variations in attitudes and preventive practices were identified. While knowledge levels did not differ significantly across sociodemographic groups, age and educational level were associated with attitudes, whereas study area and occupation were significant predictors of preventive practices. Therefore, improving toxoplasmosis prevention and control requires targeted refresher training for veterinary and human health professionals, strengthened diagnostic capacity, and enhanced collaboration between the veterinary and human health sectors through a One Health approach. These measures may improve professional preparedness and support timely diagnosis and effective disease control. Further research is warranted to identify barriers influencing knowledge, attitudes, and practices and to inform evidence‐based interventions for toxoplasmosis prevention and control.

## Author Contributions

Derara Bidira: conceptualization, resources, methodology, investigation, project administration, formal analysis, and writing–original draft.

Endrias Zewdu Gebremedhin: conceptualization, methodology, supervision, validation, formal analysis, project administration, and reviewing.

Tolesa Negasa: conceptualization, methodology, validation, supervision, and reviewing.

Isayas Asefa Kebede: software, formal analysis, validation, reference searching, and writing–review and editing.

## Funding

This research did not receive any external funding. No funding source had any role in the study design; collection, analysis, and interpretation of data; writing of the manuscript; or the decision to submit the manuscript for publication.

## Disclosure

This manuscript is an original article and has not been published elsewhere. It is not currently under consideration by any other journal or publisher.

All authors have read and approved the final version of the manuscript. Isayas Asefa Kebede, as the corresponding author and manuscript guarantor, had full access to all data in this study and takes complete responsibility for the integrity of the data and the accuracy of the data analysis.

Isayas Asefa Kebede (manuscript guarantor) affirms that this manuscript is an honest, accurate, and transparent account of the study being reported; that no important aspects of the study have been omitted; and that any discrepancies from the study as planned (and, if relevant, registered) have been explained.

## Ethics Statement

Ethical approval was obtained from the Institutional Review Board (IRB) of Ambo University (Ref. No. RCSTTVD/0005/2023). Letters of support were secured from the administrations of Holeta town, Burayu town, and Akaki Kality subcity’s agricultural and health offices. Respondents were informed about the purpose of the study, and consent was obtained from all individuals before their participation. They were informed of their right to withdraw at any time. Verbal informed consent was obtained after explaining the survey objectives and before starting the interviews, in accordance with the Declaration of Helsinki.

## Conflicts of Interest

The authors declare no conflicts of interest.

## Data Availability

The data that support the findings of this study are available from the corresponding author upon reasonable request.
